# Traditional games in elementary school: Relationships of student’s personality traits, motivation and experience with learning outcomes

**DOI:** 10.1371/journal.pone.0202172

**Published:** 2018-08-20

**Authors:** Vladimir Trajkovik, Toni Malinovski, Tatjana Vasileva-Stojanovska, Marina Vasileva

**Affiliations:** 1 Faculty of Computer Science and Engineering, Ss. Cyril and Methodius University, Skopje, Republic of Macedonia; 2 Faculty of Information and Communication Technology, FON University, Skopje, Republic of Macedonia; University of South Australia, AUSTRALIA

## Abstract

This study promotes a novel teaching approach for integration of children’s traditional games in elementary school program. It gives description of six traditional games and their educational prospects, implemented in six learning sessions in five elementary schools in Macedonia, involving 102 students. The comparison of learning achievements between these learning sessions and standard classes revealed increased students’ learning performance on comparable topics. To understand the reason for improvement, we have surveyed students after each session and tested the gathered data set via the development of a structural equation model that examines the relationships between student’s personality traits, motivation and experience with learning outcomes. The findings show that students’ achievements were directly influenced by students’ intrinsic and extrinsic motivational factors, as well as perceived experience. Additionally, the integration of traditional games in the elementary school classroom was equally accepted among all students, since their personality traits did not directly influence their experience or learning outcomes. Still, the link between the students’ personality dimensions and motivation revealed that introvert children might have slightly increased motivation and possibility to open up during game-play in such collaborative environments.

## Introduction

Latest trends in the field of education indicate shift in pedagogical approaches and teaching practices while trying to increase the engagement of the students and improve the learning process [1,2]. Promotion of collaboration and social activities among students increases involvement in learning, while sharing one’s own ideas and responding to others’ reactions sharpens thinking and deepens understanding [3–5]. Active engagement of the student following the “learning by doing” theory [6] with hands-on authentic exercise and tasks, create experiential learning environment that increases the level of students’ achievements [7–10]. Games demonstrate entertainment and commercial potential, but also can be used for “learning by doing” activities providing instructional guidelines, problem solving challenges or practical tests of individual skills [3,4,11].

Game Based Learning (GBL) has emerged from an idea to involve games in the educational process, aiming to enhance learning activities through an interesting media that captures, retains student attention and interest in subject, as well as offers intuitive and collaborative environment [12–15]. GBL scenarios engage learners into interactive, problem-solving situations that encourage critical thinking, communication, collaboration, and adaptability for functional knowledge acquisition. In addition, the game-players are usually highly motivated to engage in the gaming activities, driven by the story/goal behind, interactivity and possibility to improve performance through repetition. Hence, GBL can engage and motivate students to actively process educational content and foster development processes in the child consciousness, as well as improve experience, self-efficacy, and students' satisfaction in similar learning environments [16–18].

Despite the advantages that game playing can contribute to the learning process, there is an important gap between theory and practice for effective integration of games in the educational programs, due to different barriers for implementation [16,19–22]. It can be rather difficult to select or create proper game that can be used on a specific topic in different classes. Some studies research the use of off-the-shelf computer games that have educational potential [12,23,24], indicating that the designers of commercial computer games are not interested in providing a learning tool, but are more focused on a powerful gaming experience. On the other hand, it can be rather challenging to design and create a computer game especially for education [15,25–28], which has educational content that can be shared and globalized to cover cost charges for development. Different research suggests more integrated approach which focuses on the whole experiential environment [29,30], not just the utilized game, even though the number of studies in this regard is still scarce [31]. Additionally, even though certain skills, such as problem solving ability increase within a game, the real challenge comes when these skills and learned content have to be used outside of the gaming environment [11,32]. Some studies [32–35] have shown modest to low evidence that gamed learning skills or content can be transferred outside of the GBL environment. Thus, further research is necessary that will allow us to conclude that educational games and simulations have a positive effect on learning outcomes [16], which can be successfully replicated in everyday learning environment, as well as distinguish the important factors that influence the success of such learning process.

This study researches integration of children’s traditional games in the elementary school program, according to their potential educational value in different subjects. It analyses factors influencing learning outcomes in similar environments, such as student’s personality traits, motivation, and experience. This study is part of an ongoing project in elementary schools in Macedonia entitled “Grandma’s games”, which started in 2010 and has already included more than 10 elementary schools. The project promotes incorporation of old forgotten traditional children’s games in the everyday learning, environment enhanced with commonly available computer programs. In this study, we cover six traditional games that children, their parents, or grandparents have played while growing up in this region, which students can reuse at home and learn through game-play with their parents or classmates. Through careful preparation and development of storyline behind each traditional game according to the state program curriculum, we have incorporated standard technological tools towards creation of cognitive learning environment that can help students transfer gaming skills in the classroom for increased learning outcomes. Consequently, the study provides a description of each traditional game and activities conducted in the elementary school learning environment, as well as examines the relationships between student’s personality traits, motivation and experience with learning outcomes.

## Theoretical background

In line with the concept for discovery learning, Piaget [36] has developed a constructivist learning theory that places the learner into in the centre of educational process by promoting knowledge acquirement through active direct experience. Vygotsky [37] has shared Piaget’s assumptions for the way children learn [36] and has emphasized the importance of social interaction, while identifying games, simulations and problem-solving activities as examples of social constructivist classroom. Still, it is difficult to create a computer game for a specific educational content, which can be globalized to cover the cost for the development of such game [19,25,26]. Similarly, it is rather challenging to use available commercial computer games in the educational process [21,23,24,38], since typically their focus is on rich gaming experience and not on educational prospects. On the other hand, each region has its own traditional games, which can be used for educational purposes, while the integration of these games will shift the pedagogical approach from teacher-centered to student-centered environment [35,39,40]. Even though the literature lacks with studies that successfully couple learning design with traditional game features, researches in [41] emphasize that these games smoothen social communication and amplify the excitement caused by the game, which can be beneficial in the educational environment. Furthermore, Vasileva et al. [42] provide evidence for the usefulness of traditional games for different educational objectives, as well as their educational benefits. Similarly, in [35] a traditional game was utilized for development of computer game that was used in the classroom, which improved students’ learning experience, as well as increased effectiveness and flexibility in the class.

Malone [43] has recognized the importance of motivation during GBL and has developed a rudimentary theory of intrinsically motivating instruction. Motivated student is focused and self-determined on the educational activity without additional stimuli needed to retain his attention [16,44,45]. Students’ motivation can be either intrinsically or extrinsically driven, while both can be more effective and lasting than the other in different situations [16,25,46–48]. Intrinsic motivation refers to inner desire to engage in a task out of interest, challenge or amusement, while extrinsic refer to behaviour that is driven by external rewards, such as higher grades, social influence, etc. Still, both stimuli are significant during GBL [49–51]. Malinovski et al. [47] provide an example of intrinsic and extrinsic motivational factors, significantly linked with students’ experience in different learning environments, which demonstrate the importance of motivation during the educational process. On the other hand, even though different studies show evidence that students’ motivation and learning outcomes can be positively linked, which is also applicable during GBL [17,34, 46,49,50], via review of literature Erhel and Jamet [16] have emphasized that additional efforts are needed to provide definite relationship between level of students’ motivation and learning outcomes.

Similarly, students’ positive experience and satisfaction are significant during the educational process, since student-centered environments that fulfil users’ expectations tend to have higher learning performances [17–19]. An appropriate game integration with the curriculum that improves the students' motivation, experience, and satisfaction with the learning approach [17,52], can potentially increase the level of learning outcomes.

Integration of the GBL in the learning environment may also be influenced by students’ personality traits [53–55]. In his personality theory, Eysenck [56] recognizes three main personality dimensions: Neuroticism, Extroversion and Psychoticism. HANES methodology [57], which is an adapted version of Eysenck’s personality inventory for children and youth, evaluates the following personality traits: Neuroticism and Extroversion, which also provides two sub-traits, Sociability and Activity. Similarly, the “Big Five” model, often used in literature for personality traits evaluation [55,58,59] of varying ages, has five dimensions of personality: conscientiousness, openness to experience, extraversion, agreeableness, and neuroticism. Hence, these methods can be used to establish a link, if any, between students’ personality traits and learning performance [53,60] in different learning environments.

“Grandma’s games” project introduces a novel approach that revives children’s games played by many generations into the elementary school program, as well as leverages advantages of GBL enhanced with technology. This study embraces the social context of learning, researches concrete traditional games in elementary schools, which tend to increase students’ motivation, satisfaction, experience, and ultimately provide higher learning performance. Consequently, it analyses the relationships between intrinsic and extrinsic motivational factors [16,43,47], perceived students’ experience [17,47] and personality traits based on HANES methodology with learning outcomes during 2014/2015 school year. Hence, this study evaluates the following research questions:

- Can traditional games be successfully integrated in the elementary school classroom environment?
- Do students’ personality traits influence their motivation and experience from classes that include traditional games enhanced with technological tools?
- Is there a link between intrinsic and extrinsic motivational factors and their perceived experience in these classes?
- What effects do students’ personality traits, motivation and experience have on the learning outcomes during GBL with traditional games in the elementary school?

## Methodology

### Participants and design

This study included five K9 elementary schools in Macedonia, which were already part of the “Grandma’s games” project, and 102 students, 56 boys and 46 girls. Three schools are located in cities in Macedonia and two in villages, which provides diversity in urban and rural environments. Students were part of two groups: 53.92% were students of age 7–8 years (2^nd^ and 3r^d^ grade) and 40.08% students of age 11–12 years (6^th^ and 7^th^ grade). The involved teachers were already part of the referred project and have shared common teaching approach on selected subjects and topics according to the state primary education curriculum.

Macedonia’s region abounds of traditional games that students’ parents and grandparents played when they were children, and some of these games or variations are still popular among young population. Traditional games are non-digital games, which are played for generations as informal games without special commercial products, mostly for entertainment among children. As emphasized in Lameras et al. [61], we aimed to introduce these games into classroom, while interlinking the learning attributes to traditional game elements, so we can balance learning with gameplay. Thus, we have started with analysis of the educational prospects of particular traditional games, as well as the possible subjects and topics whose learning objectives can be achieved via GBL. For example, traditional games that involve artistic and creative activities can be used during Art classes, games with numbers and calculations during Math, as well as games with group play, social and cultural related activities during social science subjects.

The referred project already involved several primary schools and teachers for few years, which were able to use and integrate traditional games in the classrooms, as well as have tried to align them with Macedonian state curriculum. The involved teachers in the project, leveraged the feedback from students and their parents to identify number of games that have educational potential and were popular in this region in the past, so we benefited from their experience while selecting games for this research study. Traditional games can be played without reference to written rules, and the game structure is usually learn by example from other children. Thus, we could enrich certain elements (to improve calculation skills, reasoning, and memorizing, modelling and shape creation, recognize specifics of different cultures, etc.), while retaining the original design, to perpetuate learning in optimal ways [61]. Hence, students were motivated to finish the tasks in the game itself, but also performed activities according to the thematic unit in the class and learning objective.

Since traditional games are usually played with little equipment, we have also included different teachers and students ideas for technology enrichment of these games and unified them in the class. In addition, the classes with the new teaching approach used similar contexts that were covered in the regular learning process, so we could immediately realize whether the GBL with traditional games provided better results. Furthermore, to diminish the subject influence on individual students (some students may prefer one subject to another) we have covered three different subjects with and without GBL. Hence, in this study we have analysed integration of six traditional games in six learning sessions respectively: “Matchbox” and “Hop-scotch” in Math classes, “Lady” and “String” games in Art classes, “Mosque” and “Hide and Seek” games in Nature and Society classes. The chosen traditional games were visualized and graphically enhanced with technological tools like presentation software that was used to explain the games and educational goals, design applications to draw scheme designs for “Hop-scotch” on a computer and scheme of spots for “Lady”, as well as word and spreadsheet programs for presentation of results after each game. Even though, the original game design was not altered, these tools enriched the gaming and learning experience. Detailed description of each game, thematic units, and learning objectives can be found in the S1 Appendix.

### Measures and procedure

The teachers have evaluated the learning outcomes after each learning session while grading students from 1 (poor) to 5 (excellent) on a test score (L1), students’ interest (L2) and interactivity (L3). Through these performance variables, we were able to compare the learning outcomes during classical sessions and those with game-based activities.

In addition, we have evaluated several factors aiming to establish relationships with the learning outcomes, according to the theoretical background and previous experience during the project. Since it is difficult to include all influential factors in social studies, we have aimed to cover possible variations with the chosen measurement instrument and achieve high coefficient of determination for the learning outcomes. For example, even though each teacher has a unique way of teaching, children may not be comfortable or free to grade teachers’ performance, but a compounding construct that may be influenced by the teacher (like motivational factor) can cover such variations. Hence, we have evaluated the following influencing factors:

- Motivation, while distinguishing intrinsically or extrinsically driven motivational factors;
- Perceived students' experience for increased effectiveness and productivity while learning, as well as enjoyable feeling during the learning sessions;
- Personality traits according to the HANES methodology [57], as adapted version of Eysenck’s [56] personality inventory for children and youth.

Since multi-item measures are more adequate than single-item when measuring complex constructs [62], we defined a set of observed variables for each construct (complex unobserved variable). According to HANES methodology, we have used two questionnaires: HANES-1 and HANES-2 (36 and 32 questions respectively) to evaluate students’ personality traits, as well as separate survey, based on both published and researcher-developed instruments, to gather students’ opinion regarding their motivation and perceived experience (S1 Questionnaires). Table 1 depicts detailed information for the measurement instruments that was evaluated in respect to the learning outcomes.

**Table 1:** Constructs and chosen indicators used as measurement instrument.

Construct	Indicator	Description	Derived from
**Personality Traits**	P1	Sociability–talkative and cooperative in nature	[53,57]
	P2	Activity–initiative and dynamic	
	P3	Extroversion–open, fun-loving, seeking stimulation in the company of others	
	P4	Neuroticism–tendency to experience anger, worry or sadness easily	
**Motivation**	M1	Influence by the challenge and goal-oriented task	[16,25,27,47]
	M2	Beliefs for inner desire to engage in the task	
	M3	Desire for higher grades	
	M4	Obligation to actively be part of the task	
**Experience**	E1	Beliefs for increased efficiency in learning	[17,27,47,63,64])
	E2	Beliefs for increased possibilities and productivity	
	E3	The teaching approach is interesting and enjoyable	
	E4	Overall satisfaction from this type of school activities	

The survey according to HANES methodology had different questions, but the resulting classification on each personality dimension was from 1 to 9 (1 = extremely below average, 9 = extremely above average). The indicators for the motivation and experience constructs, were phrased on a five-point Likert scale [65] (1 = strongly disagree, 5 = strongly agree), retrieved as students’ self-reporting information. The teachers did not influence students’ decisions and students’ personal data and privacy was protected at all time. Hence, we have assessed the internal consistency of the surveyed items for each construct in respect to the gathered data set through Cronbach's alpha test [66]. Consequently, this study uses structural equation modeling (SEM) [67] to develop a model that adequately represents relationships between student’s personality traits, motivation, and experience with learning outcomes, based on the researched constructs.

### Ethics statement

State regulation for elementary and high schools in Macedonia lists participation in research projects that enhance teaching methods as one of teacher's responsibilities, which usually include surveys and some form of children’s evaluations that require approval from parents or guardians of the involved children. Since, there is no Ethic Committee in Macedonia that covers such matters (besides state regulations), the ethical approval for the “Grandma’s games” project, which encompasses this research study, was initially obtained from the Institutional Review Board of the Primary School "Sveti Kiril i Metodij—Centar", Skopje, Macedonia. This school started the “Grandma’s games” project and the same procedure was performed later in other schools that participated in the project, while written consent was obtained by the parents or guardians of the involved children. The consents were verified and approved by administrators of these. The researches were not present in the classes, and the study activities were covered by the referred consents, including the data analysis, which was performed anonymously.

## Results

The resulting data set was obtained from 587 responses for the involved 102 students. Most of the students participated in the six learning sessions, since only a small percentage was sick at a particular session (we obtained 4.15% less responses compared to the theoretical value if no one was sick). Therefore we were able to gather comprehensive and relevant information, which is sufficient for the SEM analysis [68, 69]. Since the classical and GBL sessions were conducted on comparable topics on all subject, we could draw immediate conclusion whether the introduction of the traditional games provided positive change for the learning outcomes.

The tests showed that integration of traditional games in the classroom environment has increased the learning outcomes. Additionally, the other two performance indicators were also higher during GBL, since collaboration and teamwork increased the level of interest and interactivity among children. To reveal the reason for the increased learning performance, we used the gathered data set for further factor analysis and development of a model representing relationships between the study’s researched constructs.

In order to improve reliability, validity, and stability of the constructs [70,71], we have performed exploratory factor analysis and removed indicators which have low loading coefficient (factor loading < 0.50) and are not statistically significant for each construct. The resulting Cronbach's alpha test for the retained indicators on respective constructs were significantly above the threshold value 0.7 [62], as evidence for strong internal consistency for the measurable items within each construct. Table 2 lists descriptive statistical information for the rest of the measures and results from the Cronbach's alpha test for each construct.

**Table 2:** Descriptive statistical information for the chosen indicators.

Construct	Indicator	Mean	SD	Cronbach's alpha
**Personality** **Traits**	P1	5.53	1.237	0.876
	P2	5.20	1.451	
	P3	5.24	1.537	
	P4*	8.36	0.726	
**Motivation**	M1	4.83	0.548	0.838
	M2	4.76	0.655	
	M3	4.83	0.495	
	M4	4.86	0.507	
**Experience**	E1	4.87	0.494	0.818
	E2	4.68	0.741	
	E3	4.80	0.577	
	E4	4.81	0.554	
**Learning Outcomes**	L1	4.83	0.559	0.764
	L2	4.85	0.498	
	L3	4.86	0.447	

*Items extracted during data analysis to achieve internal consistency within constructs.

Through development of SEM model we have analysed complex relationships between researched constructs, their behaviour and influence on learning outcomes when traditional games were integrated in the elementary schools. Hence, the model was tested for model fit indicates to validate the degree of alignment with collected data set, as suggested by previous research, with satisfactory results shown in Table 3.

**Table 3:** SEM model goodness-of-fit indices for n = 587.

Construct	Recommended value	SEM model	Source
**CMIN**	-	285.507	
**df**	-	71	
**CMIN/df**	< 5	4.021	[72]
**GFI**	> 0.90	0.938	[73,74]
**AGFI**	> 0.90	0.908	[74]
**CFI**	> 0.90	0.961	[75,76]
**NFI**	> 0.90	0.949	[73,77]
**RMSEA**	< 0.08	0.07	[78,79]

CMIN = calculated chi-square, df = degrees of freedom; GFI: goodness-of-fit index; AGFI: adjusted goodness-of-fit index; CFI: comparative fit index; NFI = normed fit index, RMSEA: root mean-square error of approximation.

**Fig 1:**
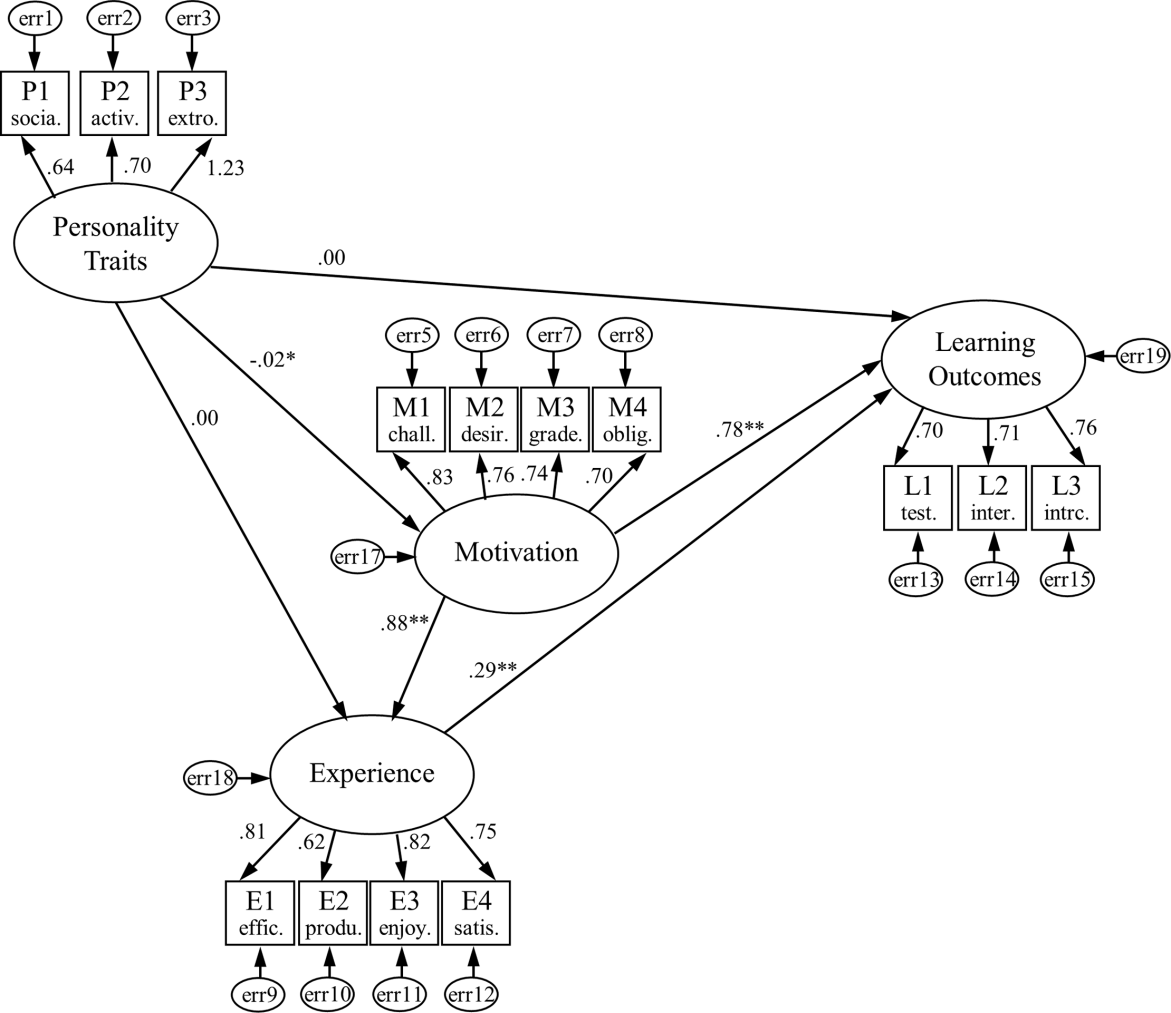
Structural equation model for relationships between student’s personality traits, motivation and experience with learning outcomes (*p<0.05 and ** p<0.001 report significant paths, two-tailed).

The model provides factor loadings for each researched construct and proposed observed indicators, as well as resulting path coefficients for relationships between constructs, with final output for the learning outcomes. It also explains measurement errors in the analysis and R^2^ for students’ learning performance during integration of traditional games in the classroom using the researched model. Fig 1 shows the resulting SEM model according to the gathered dataset, obtained factor loadings for each construct and path coefficients between constructs.

The results from the SEM model revealed the strongest influencing measure for each researched construct, more precisely P3 (extroversion) for personality traits, M1 (challenge for the goal-oriented task) for motivation, E3 (interesting and enjoyable) for experience and L3 (interactivity) for the learning outcomes. Additionally, it shows that Learning Outcomes during classes that include traditional games were mostly determined by students’ Motivation (β = 0.78, p<0.001) and directly influenced by students’ Experience (β = 0.29, p<0.001), with R^2^ = 0.80. The link between Motivation and Experience showed strong statistical significant effect (β = 0.88, p<0.001), while Personality Traits were statistically linked only with Motivation (β = -0.02, p<0.05). Therefore, students’ Personality Traits did not play an important factor in this model since the paths Personality Traits/Experience and Personality Traits/Learning Outcomes had no significant influence with β = -0.00 and p>0.05.

## Discussion and conclusion

This study promotes integration of traditional games in the classroom environment via novel teaching method in the elementary school program, as well as fosters social interaction and corporative learning, competitive spirit and friendship, which are usually inspired during the game-playing. It enhances the GBL activities with standard technological tools that do not require additional cost or time for development, as well as programing. It follows a comprehensive approach that suggest selection of traditional games, from rich folklore in the region, and their adaptation according to state program curriculum. The study reveals that game slightly adjusted and appropriately aligned with the content traditional games can be beneficial with children of varying ages (for example math arithmetic operation within same game can be adjusted in respect to children’s age) and various topics in different subjects. Most importantly, it shows that integration of traditional games in the elementary schools can provide increased learning outcomes, not just on test scores, but also in children’s interest and engagement, as well as interaction with the teacher and classmates. The practical presentations of the material through the chosen game, enables students to better understand the abstract content and use the knowledge outside of the learning environment, which is a real challenge with GBL [11,32].

Additionally, this study analyses factors influencing learning outcomes in similar environments, such as student’s personality traits, motivation, and perceived experience. The gathered data set from the involved students was statistically analysed via SEM with a resulting model that explained more than 80% of variance for the learning outcomes, which verifies the model solid measurement structure. Even though additional items can be evaluated that may potentially influence learning performance during GBL, complex SEM models with high number of estimated parameters, or manifested variables can have difficulties achieving appropriate model fit [67,68]. Researches may try to analyse the same data set with liner regression models or neural networks and fuzzy logic techniques. Still linear regression may explain lower percentage of variance or achieve worse RMSEA [53,80], while neural networks essentially use highly nonlinear models and are not applicable for this particular case [81,82]. Hence, SEM was appropriate as predictor offering insights on gather students’ data in this study and its structure, in respect with the learning outcomes.

The results from this study strongly support research articles that emphasize the importance of motivational factors during GBL [16,25,45,46,50,51,83] including ones that show evidence for close link between motivation and learning performance [27,34,44]. When traditional games were used as instructional tool, enhanced with commonly available technology, students’ intrinsic motivational factors were more statistically significant than extrinsic. The challenge behind the goal-oriented task and inner desire to engage in the collaborative activity had slightly higher effect on the motivation construct [16,45,49,50,83,84]. In line with Chen, Wang, & Lin [85] and Connolly et al. [86] the traditional game-play supplemented with collaborative activities enriched the learning experience, in terms of effectiveness, fun, and enjoyment, which had positive link with increased learning performances [17–19].

This study also revealed positive link between motivation and students’ perceived experience, as in Malinovski et al. [47]. Consequently, the integration of traditional games in the elementary school classroom was equally accepted among all students, since their personality traits did not influence their experience or learning performance. Opposite studies [54,87,88] that found a significant difference in personality traits and game-play, the traditional games and the experiential learning environment invoked only a slight link between students’ personality dimensions and their motivation. Since, this path showed statistically significant negative connection and the extrovert measure regressed highly on the personality construct, we can conclude that learning activities with traditional games can increase motivation in introvert children (lower values for the extrovert measure influence higher values for the motivation construct). Thus, they can open up with GBL and better reach their potentials in such collaborative environment.

### Practical implications

This study promotes a new idea that builds on old and forgotten children’s’ games, as one of the major source of our tradition. Even though the traditional games are not official educational tools in elementary schools Macedonia, they are used as part of a governmental initiative to stimulate teacher to enhance their teaching methods. For those teachers that already use traditional games in the classroom, the results from the study provide information for the key elements that they should approach in advance to achieve increased learning outcomes. More precisely they should focus on:

- Students’ intrinsic and extrinsic motivational factors, with a slightly higher importance of the students’ inner motivational stimuli;
- Creation of a learning environment that will increase students’ perceived experience in these classes, while providing an interesting and enjoyable feeling during GBL, as well as to facilitate increased efficiency in learning.

In addition, certain teachers in other countries may be encouraged to use traditional games and benefit from these implications. Hence, they can use these games to visualize abstract topics or leveraged them as add-ons in the standard learning environment. Such GBL activities that increase students’ motivation in class, can invoke critical thinking, boost information processing and collaborative activities, while creating experiential learning environment that increases the level of students’ achievements.

Finally, this study may open up several new avenues for research, while the findings from the statistical analysis can help educational institutions identify factors that positively influence learning performance when traditional games are chosen as instructional tool in the classroom environment.

### Limitations

Since this study is part of an ongoing project in elementary schools in Macedonia, the researchers benefited from the involved teachers’ gained experience during class preparation on different subjects using traditional games. Even more, some of the researchers were involved in the project from the beginning, so they participated in the development of the methodology over the years, which they leveraged in this study. Therefore, it can introduce certain limitations in similar theoretical and practical approaches for GBL using traditional games, since other researchers and practitioners should spend enough time and effort on proper game selection, determine its potential to provide benefits to students on specific subjects, as well as its integration according to the state curriculum.

In addition, the results for the learning outcomes were from three different subjects, to diminish the subject influence on individual students. Thus, we have used a combined learning outcomes as a total, while the utilization of traditional games in only a specific subject might provide slight difference, which should be taken into account during practical implementations.

## Supporting information

S1 Appendix (DOCX)

S1 Fig Examples for scheme designs for the game “Hop-scotch”. (TIF)

S2 Fig Pattern examples for the game “Lady”. (TIF)

S1 Questionnaires (DOCX)
