# Supplementary material for: Traditional games in elementary school: Relationships of student’s personality traits, motivation and experience with learning outcomes
Source: PLoS One. 2018 Aug 20;13(8):e0202172. doi: 10.1371/journal.pone.0202172 (PMC6101384; doi:10.1371/journal.pone.0202172)
Supplement: S1 Appendix — (DOCX) [file pone.0202172.s001.docx]

**S1 Appendix**

**“Matchbox“**

**Description**: the game uses an empty matchbox, while children draw numbers on each side. Numbers can be sequential or random (depending on children’s age) and the matchbox is placed on an edge of a table, while one half is laying on the table. A player hits the outer half of the matchbox, while the matchbox flies in the air and lands on the table. The player has to add the number shown on the upper side of the matchbox (or perform other arithmetic operation depending on children’s age) to his score. If the player miscalculates, the others subtract (or other operation) from his score. At the same time, children keep track of each number and player overall score on their computers, using a spreadsheet, which enriched the students’ experience during the game play. Additionally, students are encourage to consider which number should be obtained in the next round to give them an advantage over previous players. The game is played in fixed number of rounds (usually five) and the highest score wins. At the end, students present their results via computer presentation.

**Subject**: Math.

**Thematic unit**: arithmetic operations and calculations.

**Learning objectives**: improve calculation skills, reasoning, and memorizing.

**“Hop-scotch“**

**Description:** children draw a geometric shape divided in 8 to 10 fields a floor with a specific design, while each field is marked with a number (S1 Fig). Graphical tools are used to draw scheme designs for game on a computer, which are than reflected in practice. Hence, the technology tools provided more options for the game itself. Following, a player has to throw a flat roundly shaped stone inside of each field in ascending order. If he successfully hits the right field, he has to jump on one foot across fields to the current one. The player is not allowed to step on a line while jumping towards the current filed, nor should the stone fall on a line. If he successfully reaches the field, its number is added (or other arithmetic operation depending on children’s age) to his score, while the children keep track of the results on their computers. The player has to cover all fields.

**Subject:** Math.

**Thematic unit:** arithmetic operations and calculations.

**Learning objectives:** improve calculation skills, reasoning, and memorizing.

**S1 Fig. Examples for scheme designs for the game “Hop-scotch”*.***

**“Lady“**

**Description:** children use computers to prepare different shapes for the game in advance. Hence, they draw the scheme of spots on a paper (S2 Fig depicts few examples), while two players compete, each one having nine pebbles, marked either red or black. The players move pebbles one by one and try to sort three pebbles in a horizontal or vertical position according to the scheme and create checkers (“lady”). The player who succeeds to create “lady” has the right to take one of the opponent’s pebbles, until the winner succeeds to take seven of the opponent’s pebbles, since the opponent cannot continue to compete with remaining two pebbles.

**Subject:** Art.

**Thematic unit:** pattern modeling and shape creation.

**Learning objectives:** visualization, developing sense for symmetry and shapes.

**S2 Fig. Pattern examples for the game “Lady”*.***

**“String“**

**Description**: the game is played with a hemp thread of approximately 60 cm, with ends tight in a knot. The goal is to create most imaginative shape by twisting and turning the string around the player’s fingers. The shapes are presented to the teacher and other students, while children take pictures and digitize the result of their efforts. The combination between the traditional game and the technology inspires the children to be more creative and inventive. Final outcomes are presented and graded electronically.

**Subject**: Art.

**Thematic unit:** symmetry, two and three dimensional projections.

**Learning objectives**: acquire symmetry concepts, parallel and diagonal lines, as well as different projections.

**“Mosque“**

**Description:** A square is drawn on the floor, while five flat round stones are placed in the center one on a top of each other, forming a tower of stones. The players are divided in two groups. The first group tries to tear down the stack of stones with a ball from the 2–3m distance. When they tear the stack, they have to rebuild it over again, while the second group tries to prevent the players to rebuild the tower by hitting them with the ball. If they manage to hit a player from the first group with the ball, that player has to leave the game. The team that manages to rebuild the tower in the scheduled time wins the game. The children keep track of the results of both groups on their computers and calculate the final scores after several rounds.

**Subject:** Nature and Society.

**Thematic unit:** understand the individual differences, cope with trilling or negative emotions, as well as develop collaboration spirit.

**Learning objectives:** handle different emotions such as empathy, friendship, trust, as well as anger, conflict, discomfort.

**“Hide and Seek“**

**Description:** the children form a circle holding their hands together, while children are preferable dressed in different folklore clothing. Previously, children take pictures of all players in the folklore clothing and thus create a digital database that each player has to remember in advance. The current player is placed in the middle of the circle with a cloth covering his eyes, while the rest sing and dance moving the circle around. When the circle stops, the player has to move towards random player from the circle and tries to guess his name by using his senses for touch and smell, as well as his clothing.

**Subject:** Nature and Society.

**Thematic unit:** recognize similarities and differences in individuals coming from different cultures.

**Learning objectives:** familiarization with different traditions and cultures in our country, encourage friendship regardless of the cultural differences.
