# Supplementary figures and images for: Traditional games in elementary school: Relationships of student’s personality traits, motivation and experience with learning outcomes
Source: PLoS One. 2018 Aug 20;13(8):e0202172. doi: 10.1371/journal.pone.0202172 (PMC6101384; doi:10.1371/journal.pone.0202172)

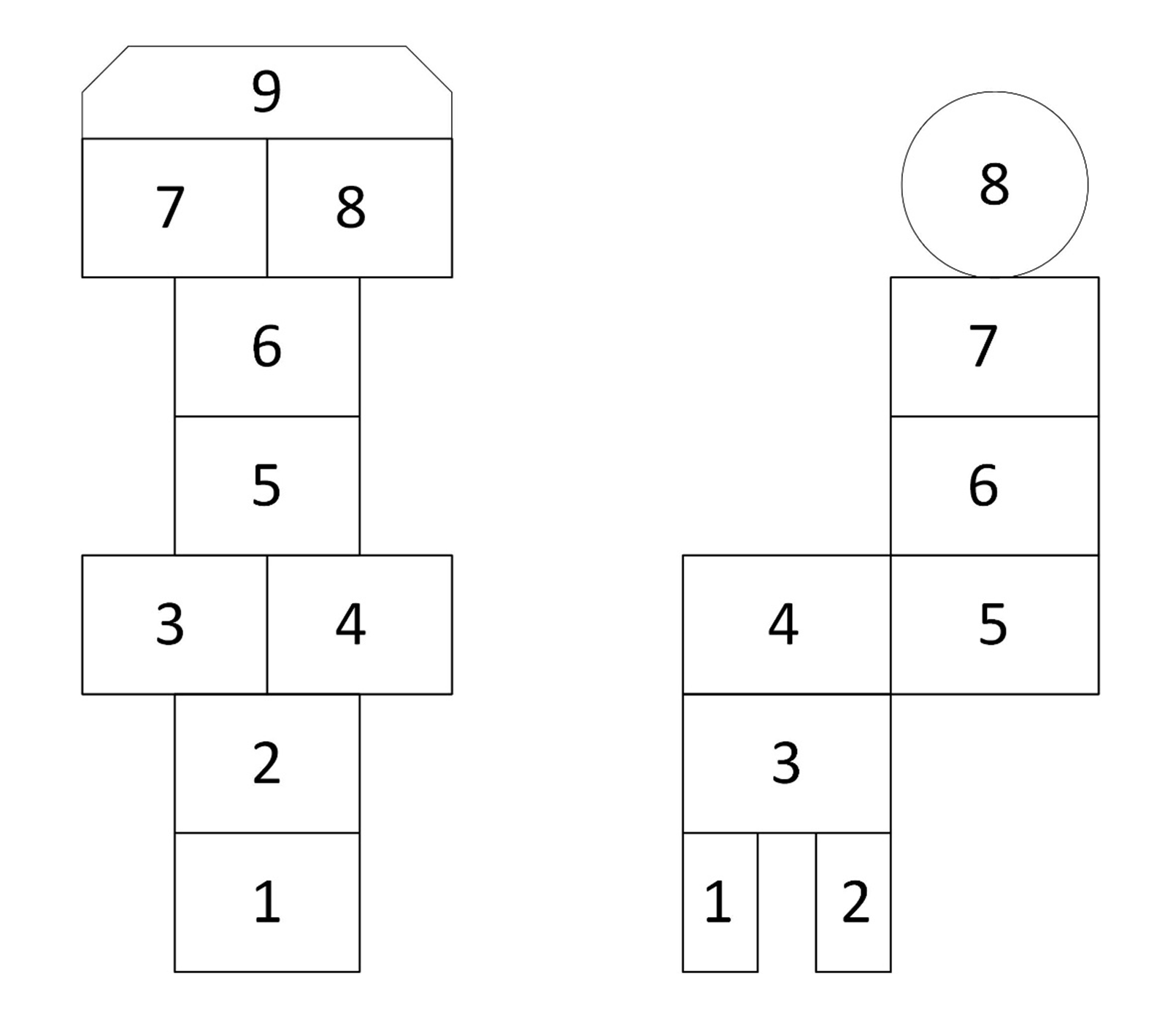

Supplement: S1 Fig — (TIF) [file pone.0202172.s002.tif]

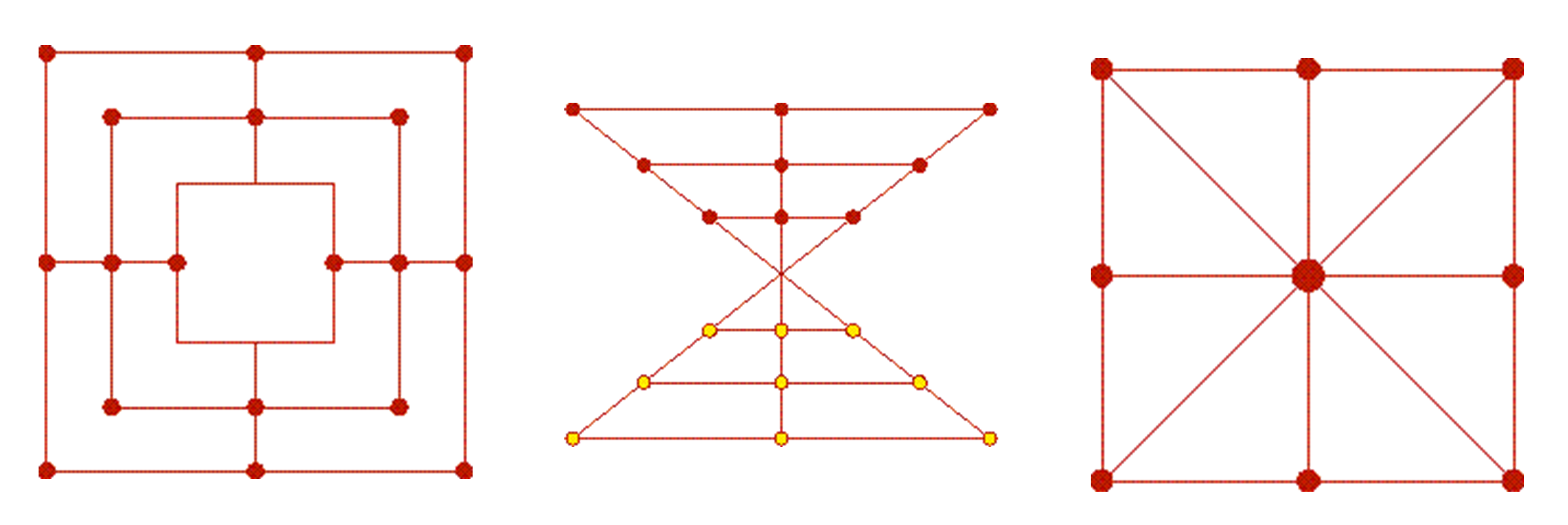

Supplement: S2 Fig — (TIF) [file pone.0202172.s003.tif]
