# Supplementary material for: Traditional games in elementary school: Relationships of student’s personality traits, motivation and experience with learning outcomes
Source: PLoS One. 2018 Aug 20;13(8):e0202172. doi: 10.1371/journal.pone.0202172 (PMC6101384; doi:10.1371/journal.pone.0202172)
Supplement: S1 Questionnaires — (DOCX) [file pone.0202172.s004.docx]

**S1 Questionnaires**

**HANES Questionnaires**

HANES 1

| 1 | I have a trembling sensation in the stomach before important events. | Yes | No |
| --- | --- | --- | --- |
| 2 | It is hard for me to be really cheerful at fun and playful birthday parties | Yes | No |
| 3 | I often think about things that I am not allowed to do or say | Yes | No |
| 4 | I like to be in the company of others | Yes | No |
| 5 | I am really sensitive about certain things | Yes | No |
| 6 | I prefer to sit and observe others at birthday parties, rather than to participate. | Yes | No |
| 7 | I sometimes have difficulties to fall asleep easily, because different thoughts run through my mind. | Yes | No |
| 8 | I like it when I am in a fun happening. | Yes | No |
| 9 | I am often fed up with everything. | Yes | No |
| 10 | I would be unhappy if I rarely have company. | Yes | No |
| 11 | I often daydream at school and at home | Yes | No |
| 12 | I am generally in a good mood. | Yes | No |
| 13 | I worry about certain past or future unpleasant events for a longer period | Yes | No |
| 14 | I am usually silent in company of others | Yes | No |
| 15 | I often believe that others are more successful than me | Yes | No |
| 16 | I often have bad dreams | Yes | No |
| 17 | I prefer to do things alone, than with others | Yes | No |
| 18 | I am often upset and I want to do something about it, but I not sure what | Yes | No |
| 19 | I often need fun friends to cheer me up | Yes | No |
| 20 | I often want to experience something fun and exciting | Yes | No |
| 21 | I am very sad when I ask for something and I am refused | Yes | No |
| 22 | I often have uneasy conscience | Yes | No |
| 23 | I often tell my friends jokes and funny stories | Yes | No |
| 24 | I often feel tired and sleepy without any specific reason | Yes | No |
| 25 | I like to take risks, even when the outcome is not certain | Yes | No |
| 26 | I often feel pain in my body | Yes | No |
| 27 | I very active and alive child | Yes | No |
| 28 | I often feel lonely | Yes | No |
| 29 | I am often in a mood for jokes | Yes | No |
| 30 | I think I am sensitive and restless child | Yes | No |
| 31 | I like to take part in wild and harsh games | Yes | No |
| 32 | I am very hurt by remarks towards me or things I do | Yes | No |
| 33 | I think I am cheerful and careless child | Yes | No |
| 34 | I have trouble falling asleep at night | Yes | No |
| 35 | I can easily lighten up a boring birthday party | Yes | No |
| 36 | I sometimes get tired even though I have not performed difficult activities | Yes | No |

HANES 2

| 1 | I often believe I could do better, when I do something important | Yes | No |
| --- | --- | --- | --- |
| 2 | I often think about my life and passed days | Yes | No |
| 3 | I always wash my hands before meals | Yes | No |
| 4 | I sometimes feel restless and cannot sit still for a longer period | Yes | No |
| 5 | I am often worried | Yes | No |
| 6 | I sometimes speak badly about others | Yes | No |
| 7 | I am often worried something bad can happen | Yes | No |
| 8 | I am nervous when I have to talk to a someone I do not like | Yes | No |
| 9 | I tend to eat everything on my plate during meals | Yes | No |
| 10 | I often feel little offended | Yes | No |
| 11 | I am angry at myself for a longer period, when I am ashamed | Yes | No |
| 12 | I immediately do what I am told | Yes | No |
| 13 | I am often in a bad mood | Yes | No |
| 14 | I sometimes like to act important | Yes | No |
| 15 | I have difficulties falling asleep if I was mad before | Yes | No |
| 16 | I always behave well, so none can object to me | Yes | No |
| 17 | I often have problems because of my recklessness | Yes | No |
| 18 | I would declare everything at customs, even when I am sure I will not be randomly checked | Yes | No |
| 19 | I sometimes feel unhappy with no specific reason | Yes | No |
| 20 | I sometimes misbehave with my parents | Yes | No |
| 21 | I am sometimes happy than sad with no specific reason | Yes | No |
| 22 | I am worried for my health | Yes | No |
| 23 | I sometimes prolong things I can to do today for tomorrow | Yes | No |
| 24 | I am easily confused | Yes | No |
| 25 | I sometimes believe life is not worth living | Yes | No |
| 26 | I am sometimes late at school or meeting | Yes | No |
| 27 | I often feel tired with not specific reason | Yes | No |
| 28 | I immediately reply to letters/emails after I have read them | Yes | No |
| 29 | I sometimes start to tremble with no specific reason | Yes | No |
| 30 | I sometimes do not tell the truth | Yes | No |
| 31 | I often have headaches | Yes | No |
| 32 | I think I am a nervous child | Yes | No |

**Questionnaire for students’ motivation and perceived experience during GBL with traditional games**

Legend


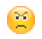

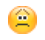

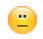

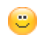

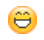


strongly disagree disagree neutral agree strongly agree

| **Please circle one emoji for each question to express your motivation:** | |
| --- | --- |
| 1. I feel challenged to solve tasks using game-play | 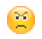 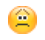 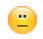 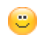 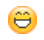 |
| 2. I am internally motivated to engage in game-based learning tasks | 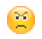 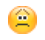 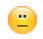 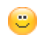 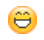 |
| 3. I believe I can achieve higher grades | 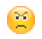 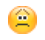 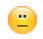 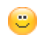 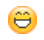 |
| 4. I am obligated to actively be part of the task during game-based learning | 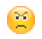 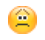 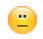 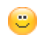 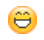 |

|  | |
| --- | --- |
| **Please circle one emoji for each question to express your experience:** | |
| 1. I believe I am more efficient and can solve tasks more quickly | 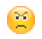 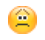 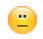 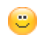 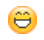 |
| 2. I believe the games in classes increase possibilities for learning and productivity | 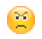 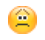 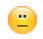 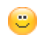 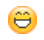 |
| 3. I think this teaching approach is interesting and enjoyable | 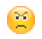 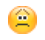 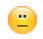 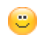 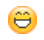 |
| 4. Overall, I am satisfied from this type of school activities | 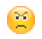 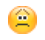 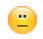 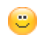 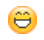 |
